# Dissection of the multigenic wheat stem rust resistance present in the Montenegrin spring wheat accession PI 362698

**DOI:** 10.1186/s12864-018-4438-y

**Published:** 2018-01-22

**Authors:** Jason D. Zurn, Matthew N. Rouse, Shiaoman Chao, Meriem Aoun, Godwin Macharia, Colin W. Hiebert, Zacharias A. Pretorius, J. Michael Bonman, Maricelis Acevedo

**Affiliations:** 1 Department of Plant Pathology, North Dakota State University, Fargo, ND USA; 2 USDA-ARS, National Clonal Germplasm Repository, Corvallis, OR USA; 3 USDA-ARS, Cereal Disease Laboratory, and Department of Plant Pathology, University of Minnesota, St. Paul, MN USA; 4 USDA-ARS, Cereal Crops Research Unit, Fargo, ND USA; 5 Kenya Agricultural and Livestock Research Organization, Njoro, Kenya; 6 Agriculture and Agri-Food Canada, Morden, MB Canada; 7 Department of Plant Sciences, University of the Free State, Bloemfontein, South Africa; 8 USDA-ARS, Small Grains and Potato Germplasm Research Unit, Aberdeen, ID USA; 9 International Programs, College of Agriculture and Life Sciences, Cornell University, Mann Library B-75, Ithaca, NY 14853 USA

**Keywords:** Food security, Yield protection, Mesothetic resistance, Infinium, SNP, KASP

## Abstract

**Background:**

Research to identify and characterize stem rust resistance genes in common wheat, *Triticum aestivum*, has been stimulated by the emergence of Ug99-lineage races of the wheat stem rust pathogen, *Puccinia graminis* f. sp. *tritici* (*Pgt*), in Eastern Africa. The Montenegrin spring wheat landrace PI 362698 was identified as a source of *Pgt* resistance. This accession exhibits resistance to multiple Ug99-lineage and North American *Pgt* races at seedling and adult-plant stages. A recombinant inbred population was developed by crossing the susceptible line LMPG-6 with a single plant selection of PI 362698. A genetic map was constructed using the Illumina iSelect 90 K wheat assay and the markers csLv34, NB-LRR3, and wMAS000003 and quantitative trait locus (QTL) analysis was performed.

**Results:**

QTL analysis identified five significant QTLs (α = 0.05) on chromosomes 2B, 3B, 6A, 6D, and 7A associated with wheat stem rust resistance. The QTL on chromosome 3B was identified using both field data from Kenya (*Pgt* Ug99-lineage races) and seedling data from *Pgt* race MCCF. This QTL potentially corresponds to *Sr12* or a new allele of *Sr12*. The multi-pathogen resistance gene *Sr57* located on chromosome 7D is present in PI 362698 according to the diagnostic markers csLv34 and wMAS000003, however a significant QTL was not detected at this locus. The QTLs on chromosomes 2B, 6A, and 6D were identified during seedling trials and are thought to correspond to *Sr16*, *Sr8a*, and *Sr5*, respectively. The QTL identified on chromosome 7A was detected using MCCF seedling data and may be *Sr15* or a potentially novel allele of recently detected Ug99 resistance QTLs.

**Conclusions:**

The combination of resistance QTLs found in PI 362698 is like the resistance gene combination present in the broadly resistant cultivar Thatcher. As such, PI 362698 may not be a landrace as previously thought. PI 362698 has been crossed with North Dakota wheat germplasm for future breeding efforts. Additional work is needed to fully understand why the combination of genes present in PI 362698 and ‘Thatcher’ provide such durable resistance.

**Electronic supplementary material:**

The online version of this article (10.1186/s12864-018-4438-y) contains supplementary material, which is available to authorized users.

## Background

The emergence of new races of the wheat stem rust pathogen, *Puccinia graminis* Pers.:Pers. f. sp. *tritici* (*Pgt*), has historically been a problem in many wheat (*Triticum aestivum* L.) producing regions [1–5]. *Pgt* is a macrocyclic rust that mainly persists in its asexual uredinial state and is primarily controlled using resistance genes [1–4]. The use of resistance genes has been particularly effective in the United States due to low pathogen diversity caused by the removal of the alternate host of *Pgt,* the common barberry (*Berberis vulgaris* L.), which is required for sexual recombination [2, 6]*.* Despite the reduction of *Pgt’s* sexual cycle in most of North America, new *Pgt* races occasionally emerge due to a sexual population in the Pacific Northwest and selection pressures imposed upon the *Pgt* population by deployed resistance genes [6]. In Eastern Africa new races also emerge due to selection pressures imposed by deployed resistance genes; however, the rate of emergence is enhanced due to multiple cropping seasons in a year causing a “green bridge” [3, 7].

The emergence of Ug99-lineage races and new virulent races in the durum [*Triticum turgidum subsp. durum* (Desf) Husn] producing regions of Ethiopia has instilled new concerns regarding global food security [3–5, 8, 9]. In 1998 the *Pgt* race TTKSK (Ug99), which is virulent on *Sr31*, was identified in Uganda [10]. Two years later a second *Pgt* race, TTKSF, was identified in South Africa with shared ancestry to Ug99 [7]. Selection pressure imposed by deployed resistance genes has resulted in Ug99-lineage races that are virulent on *Sr24*, *Sr36*, *Sr9h*, and *SrTmp* [3, 11–14]. In Ethiopia, cultivars with resistance to Ug99-lineage races were deployed [8, 9]. These cultivars led to selection against Ug99-lineage races and a predominance of the race TKTTF in these regions, which lead to major epidemics in 2013 and 2014 [9].

Over 60 wheat stem rust resistance genes have been found in wheat, at least 31 of which confer resistance to at least one race in the Ug99-lineage [3–5, 14, 15]. Approximately one third of all stem rust resistance genes and half of the resistance genes effective to Ug99-lineage races were introgressed from wild wheat relatives [5, 14]. Many of these genes suffer from linkage drag and low efficacy when deployed alone [14]. Due to the continued emergence of virulent *Pgt* races it is important to identify and characterize new sources of resistance.

Landraces can serve as unexploited sources of resistance to many diseases. The deployment of previously used resistance sources in modern breeding programs has created diversity bottlenecks for many crop species, creating vulnerability to disease [16]. As such, the increased diversity found in landraces due to their association with traditional farming systems make them useful tools for many wheat breeding programs [17–20]. Resistance genes identified in landraces are easier to incorporate into adapted material than those from wild relatives [18]. The identification and subsequent mapping of disease resistance loci from landraces has been effectively applied to wheat [21–24], including wheat stem rust resistance loci [25–28].

During a recent study to identify *Pgt* resistance in spring habit *T. aestivum* landraces, Newcomb et al. [29] evaluated 2509 accessions from the USDA National Small Grains Collection at the International Stem Rust Nursery at the Kenyan Agricultural and Livestock Research Organization in Njoro, Kenya. A total of 278 accessions resistant to Ug99 were identified, one of which being the accession PI 362698. PI 362698 was collected from Montenegro in 1971 and displayed high resistance to the Ug99 lineage *Pgt* race TTKST in adult-plant field trials and to race TTKSK in seedling tests at the USDA-ARS Cereal Disease Laboratory in St. Paul, MN, U.S.A. PI 362698 had a mesothetic infection type that ranged from ‘;1’ to ‘31;’ on seedlings and in adult-plant trials disease severity ranged from 0 to 10% with resistant (R) to moderately susceptible (MS) infection responses [29–31]. A mesothetic infection type is unusual but a similar mesothetic seedling infection type has been observed for ‘Thatcher’, which is postulated to have the resistance genes *Sr5*, *Sr9g*, *Sr12*, and *Sr16* [32–34]. PI 362698 is postulated to have *Sr57* based on DNA markers, however any additional resistance genes present within the line were unknown [29]. To evaluate the mode of inheritance for TTKSK resistance a single plant selection of PI 362698, PI 362698–1, was crossed to the susceptible Canadian line LMPG-6 (Little Club//Prelude/8*Marquis/3/Gabo) [30, 35]. Mendelian ratios corresponding for one to three genes were not observed during seedling resistance evaluations at the F_2_ generation, suggesting the stem rust resistance to TTKSK found in PI 362698–1 is complex [30]. Consequently, the LMPG-6/PI 362698–1 population was advanced via single seed descent to identify the genetic regions associated with stem rust resistance in PI 362698–1.

## Methods

### Population development

The susceptible Canadian line LMPG-6 [34] was used as a female parent and crossed to PI 362698–1, a single plant selection [30]. A recombinant inbred population was created by advancing the F_2_ progeny via single seed descent to the F_6_ generation. Seeds from the 151 F_7_ generation individuals were pooled and advanced to produce enough seeds for phenotyping.

### Phenotypic evaluation

The LMPG-6/PI 362698–1 population, PI 362698, and PI 362698–1 were tested for seedling resistance to North American (NA) and African *Pgt* races. PI 362698 was tested against the NA *Pgt* races MCCFC (isolate 59KS19), QFCSC (06ND76C), QTHJC (75D717C), RCRSC (77ND82A), RKQQC (99KS76A), TPMKC (74MN1409), and TTTTF (01MN84A-1-2) and the African *Pgt* races TRTTF (06YEM34–1), TTKSK (04KEN156/04), and TTKST (06KEN19v3) to determine the spectrum of seedling resistance present in PI 362698. PI 362698–1 was tested against the NA *Pgt* races HHBJ, HKHJ, HKQJ, HPCJ, HPLB, QFCQC, QKCS, QKMS, QTHJ and the African *Pgt* races TTKSF and TTKSF+. Races MCCFC, QFCSC, QTHJC, RCRSC, RKQQC, TPMKC, TTTTF, TRTTF, TTKSK, and TTKST were evaluated at the Cereal Disease Laboratory. Races TTKSF and TTKSF+ were evaluated at the University of the Free State in Bloemfontein, South Africa and races HHBJ, HKHJ, HKQJ, HPCJ, HPLB, QFCQC, QKCS, and QKMS were evaluated at North Dakota State University in Fargo, ND, U.S.A. The LMPG-6/PI 362698–1 population was tested for seedling resistance against *Pgt* race HHBJ (isolate R29J), HPLB (A-15), MCCF (A-5), and QFCQC at North Dakota State University; TRTTF at the Cereal Disease Laboratory; and TTKSF+ at the University of the Free State. Two replicates of the population were planted with five seeds per experimental unit. Seedling evaluations at the Cereal Disease Laboratory were conducted by inoculating seedlings 7 to 10 days after planting with urediniospores retrieved from −80 °C storage. Urediniospores were revitalized with a 15 min 45 °C heat shock followed by 2 to 4 h rehydration under 80% relative humidity created with a KOH solution [36]. A spray inoculator was used to distribute a urediniospore and mineral oil suspension (Sotrol 170, Philips Petroleum, Borger, TX, U.S.A.). The oil was allowed to evaporate from the plants in a fume hood for 30 min and then plants were placed in a dark dew chamber for 14 h at 18 °C after which the plants were exposed to fluorescent light for 3 to 4 h and transferred to a 18 ± 2 °C greenhouse with a 16 h photo period [36, 37]. Inoculations at North Dakota State University and University of the Free State were similar to those conducted at the Cereal Disease Laboratory; however freshly collected *Pgt* urediniospores were used and greenhouse temperatures ranged from 20 to 24 °C. The plants were grown for 12 to14 days before evaluating infection types using the 0 to 4 scale developed by Stakman et al. [31].

The LMPG-6/PI 362698–1 population was tested for field response to Ug99-lineage races during the 2014 main season, July to October, and the 2015 off season, February to May, at the International Stem Rust Nursery at the Kenyan Agricultural Research and Livestock Organization in Njoro, Kenya. Hill plots with 10 to 15 seeds were planted between spreader rows of several wheat lines, including ‘Cacuke’, bearing *Sr31* and *Sr24* to select for *Pgt* race TTKST. The population was replicated twice with the parental lines planted every 20 entries to evaluate the distribution of disease pressure. A mixture of talc powder and urediniospores was used to inoculate the spreader rows. The plants were rated using categorical scores to evaluate infection response and the area of infection using a modified Cobb Scale ranging from 0 to 100% [38, 39]. Infection response categories could be used individually or in combination to describe multiple responses on a stem. The categories included immune (I), resistant (R), moderately resistant (MR), moderately susceptible (MS), and susceptible (S) [39].

### Linkage mapping

Leaf tissue was collected from each recombinant inbred line (RIL) at the F_6_ generation, lyophilized for 24 h, and then ground using a Retsch mm301 mixer mill (Retsch GmbH, Haan, Germany) as described in Rouse et al. [40]. DNA was extracted in 96-welled plates using a modified cetyltrimethylammonium bromide (CTAB) method [41]. The F_6_ RILs and parental lines were genotyped using the Illumina Infinium iSelect 90 K wheat SNP assay [42], an Illumina BeadStation, and an Illumina iScan according to the manufacturer’s protocol (Illumina Inc., San Diego, CA, U.S.A.). The markers were scored using version 1.0 of the polyploid clustering module for Illumina GenomeStudio^®^ version 1.9.4 (Illumina Inc.).

The diagnostic STS marker csLv34 was used to map the cloned resistance gene *Sr57* [43]. Reactions were completed at a volume of 20 μl containing 1X PCR buffer, 2.5 mM MgCl_2_, 187.5 μM dNTPs, 500 nM primer, 60 ng DNA, and 1 unit of GoTaq (Promega Corporation, Madison, WI, U.S.A.). Amplification was performed under the following conditions: initial denaturation at 94 °C for 5 min, followed by 40 cycles of a 30 s denaturation at 94 °C, a 57 °C annealing step for 30 s, and an extension step of 72 °C for 45 s, followed by a final extension at 72 °C for 7 min. Amplicons were visualized via 2% agarose gel electrophoresis.

The KASP markers NB-LRR3 [32] and wMAS000003 [44] were included for mapping due to their associations with *Sr12* and *Sr57*, respectively. *Sr12* often displays a mesothetic response to many races at seedling stages [32]. wMAS000003 is designed to detect the functional polymorphism for the resistant allele of *Sr57* [44]. The KASP assays were performed as 5 μL reactions consisting of 2.5 μL of KASP 2X reaction mix (LGC Ltd., Teddington, United Kingdom), 0.07 μL of KASP primer mix (containing 12 μM of each allele-specific forward primer and 30 μM reverse primer diluted in 10 mM TRIS, pH 8.3), and 37.5 ng of DNA. Amplifications were performed under the following conditions: initial denaturation at 94 °C for 15 min, followed by 10 cycles of a 20 s denaturation at 94 °C and a combined annealing and extension touch-down step for 1 min where the initial temperature began at 61 °C and decreased by 0.6 °C per cycle to reach a final temperature of 55.6 °C, followed by 26 cycles of denaturation at 94 °C for 1 min and annealing and extension at 55 °C for 1 min. Reaction fluorescence was measured using an Omega Fluorostar plate reader (BMG LABTECH GmbH, Ortenberg, Germany). Allelic data were analyzed using KlusterCaller version 2.21 (LGC Ltd).

ISelect markers with less than 5% missing data, NB-LRR3, wMAS000003, and csLV34 were used for mapping. Mapping was conducted using Mapdisto version 1.7.7.0.1.1 using a LOD of 5 and maximum recombination frequency of 0.1 [45]. The robustness of the map was evaluated using a ripple command with a window size of five markers. Problematic loci were identified using the drop command and all markers which increased the map length more than 3 centimorgans (cM) were removed. The ripple command was applied after each problematic locus was removed to revalidate the robustness of the map. Linkage groups were assigned to chromosomes using the 90 K wheat consensus map [42] and combined into chromosomal linkage groups with gaps less than 35 cM. The genetic distance between markers was calculated using the Kosambi mapping function [46].

### QTL analysis

To utilize the data for quantitative trait locus (QTL) analysis, adult-plant infection responses were converted to a numerical value modified from Yu et al. [47]. The disease responses immune (I), resistant (R), resistant-moderately resistant (RMR), moderately resistant (MR), intermediate (MRMS), moderately susceptible (MS), moderately susceptible-susceptible (MSS), and susceptible (S) were converted to the ordinal values 0, 0.2, 0.3, 0.4, 0.6, 0.8, 0.9, and 1, respectively. Infection coefficients were calculated by multiplying the numerical disease response by the severity percentage [47]. Seedling infection types were converted from the 0 to 4 Stakman scale to a 0 to 9 linear scale as described by Zhang et al. [48]. Median values were calculated for replicates and used for QTL analysis. The Shapiro-Wilk test was used to determine the normality of the population data. All statistical tests were performed using R version 3.3.2 [49]. QTL analysis was conducted using QGene version 4.4.0 [50]. Traits were analyzed using a multiple interval mapping algorithm based on a general linearized framework (MIM-GLZ) [51]. Significant QTLs (α = 0.05) were identified using a resampling analysis of 1000 permutations [52]. The 95% confidence intervals for significant QTLs were estimated using the 2-LOD drop method [53]. The Kruskal-Wallis test was used to test for epistatic interactions. Post-hoc analysis was conducted using Student’s T-Test with the Benjamini-Hochberg correction for multiple comparisons.

## Results

### Phenotypic evaluation

**Fig. 1:**
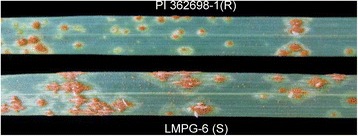
Typical mesothetic seedling resistance displayed by PI 362698–1 compared to the susceptible line LMPG-6. Infection types from the African *Pgt* race TTKSF are shown

PI 362698 was resistant to the races tested at seedling stages and was immune to many of the NA *Pgt* races (Table 1). The races TPMKC, TTKSK, TTKSF, and TTKSF+ exhibited mesothetic reactions at seedling stages (Fig. 1). Infection types varied greatly from race to race when evaluating the LMPG-6/PI 362698–1 population with races HHBJ, HPLB, MCCF, QFCQC, TRTTF, and TTKSF+ at the seedling stages (Table 2). Missing data was observed in each of the trials. In the seedling trials 13 (8.6%), 23 (15.2%), 23 (15.2%), 24 (15.9%), 27 (17.9%), and 32 (21.2%) individuals had missing data for both replicates for the *Pgt* races QFCQC, TRTTF, TTKSF+, HHBJ, HPLB, and MCCF, respectively, due to poor seed quality of some of the individuals. During the 2014 Kenyan adult-plant trial 28 (18.5%) individuals were missing between both replicates. The number of missing individuals between both replicates during the 2015 Kenyan adult-plant was higher, 48 (31.8%), due to a drier than average environment during the early part of the growing season. Biological replicates for each trial were consistent and replications were not statistically different from one another when analyzed as matched pairs (α = 0.05).

**Table 1:** Median seedling infection types (IT) of PI 362698 when inoculated with North American and African *Pgt* races

Race	Origin	Median IT
HHBJ	N. America	0
HKHJ	N. America	0
HKQJ	N. America	0
HPCJ	N. America	0
HPLB	N. America	0
MCCF	N. America	0/;
QFCQC	N. America	22−/2
QFCSC	N. America	0
QKCS	N. America	;3
QKMS	N. America	;12
QTHJC	N. America	0
RCRSC	N. America	0
RKQQC	N. America	0/1
TPMKC	N. America	;3
TTTTF	N. America	0
TRTTF	Africa	2-
TTKSK	Africa	;3
TTKSF	Africa	;3
TTKSF+	Africa	;3
TTKST	Africa	0

**Table 2:** Seedling infection types and adult disease infection responses (IR) and severities for PI 362698–1, LMPG-6, and the LMPG-6/PI 362698–1 population

Seedling Trial	PI 362698–1		LMPG-6		LMPG-6/PI 362698–1	
HHBJ	0		33+		0 to 3 + 4	
HPLB	0		33+		0 to 3 + 3	
MCCF	0		3 to 33+		0 to 4	
QFCQC	2–1 to 2		3 to 34		1 to 3 + 4	
TRTTF	12- to 2-		3+		;12- to 3+	
TTKSF+	;123		3+		;12 to 4	
Adult Trial	PI 362698–1		LMPG-6		LMPG-6/PI 362698–1	
	IR	Severity	IR	Severity	IR	Severity
Kenya 2014	I to R	0% to Trace	MS to S	20 to 60%	R to S	Trace to 60%
Kenya 2015	R to MR	0% to Trace	MSS to S	40 to 70%	R to S	Trace to 70%

Seedling infection type scaled ranges from 0 to 4 and observations with multiple infection types are listed in order of frequency. Plus (+) and minus (−) signs were used to indicate increased or decreased sporulation for an infection type. Adult infection responses are classified as immune (I), resistant (R), moderately resistant (MR), moderately susceptible (MS), and susceptible (S). Intermediate infection responses are described by recording both responses observed. Adult severity ratings can range from 0% to 100% and trace severities indicates severities between 0% and 5%

### Linkage mapping

The STS marker csLv34, KASP markers NB-LRR3 and wMAS000003, and 6863 SNP markers from the 90 K Infinium iSelect wheat assay were used for mapping. A genomic map with a total length of 2544.3 cM was produced consisting of 2153 unique loci (bins) distributed across 32 linkage groups (Additional file 1: Table S1). Co-segregating markers were found at 44.3% of the bins, ranging from 2 to 65 co-segregating markers per bin. Severe segregation distortion was observed for the long arm of chromosome 3B, approximately a 3:1 ratio of PI 362698–1 to LMPG-6 alleles for each locus, and linkage group 5A2, approximately a 2:1 ratio of PI 362698–1 to LMPG-6 alleles at each locus. Markers at these genetic locations were homozygous in each parent.

### QTL analysis

**Fig. 2:**
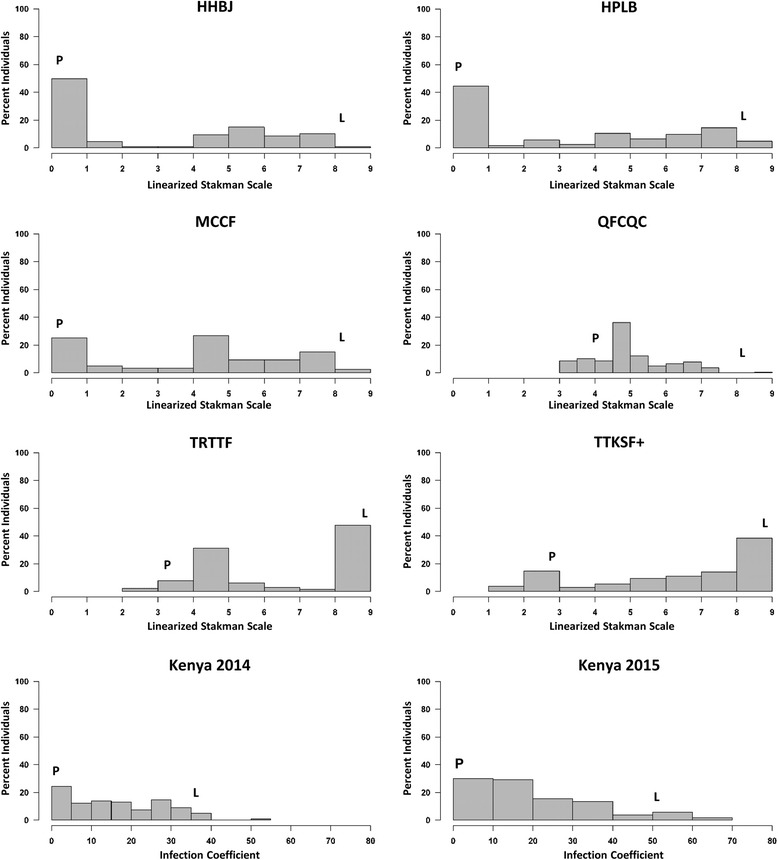
Distributions of the seedling (HHBJ, HPLB, MCCF, QFCQC, TRTTF, and TTKSF+) and field (Kenya 2014 and Kenya 2015) data for the LMPG-6/PI 362698–1 population. Distribution is expressed as the percent individuals within a phenotypic category. Median phenotypes for PI 362698–1 and LMPG-6 are designated with P and L, respectively

All converted data from the seedling and adult-plant trials were not normally distributed (α = 0.05; Fig. 2). Data from the seedling trials revealed five significant QTLs (α = 0.05) on linkage groups 2B, 3B, 6A, 6D1, and 7A2 (Table 3). Each of these QTLs explained greater than 34% of the phenotypic variation for their respective trials, with the resistant alleles being inherited from PI 362698–1. No significant QTLs (α = 0.05) were observed for *Pgt* race TTKSF+. Non-significant QTLs for TTKSF+ were observed near the 2B (*QSr.ace-2B*), 3B (*QSr.ace-3B*), 6A (*QSr.ace-6A*), and 7A2 (*QSr.ace-7A2*) QTLs. *QSr.ace-3B*, which was detected during the MCCF seedling trial, was detected during both field trials in Kenya during 2014 and 2015 (Table 3). This QTL explained 47% and 50% of the phenotypic variation for the 2014 and 2015 Kenyan trials, respectively. The position of this QTL mapped near the *Sr12* associated marker NB-LRR3 but shifted slightly between each of the trials, likely due to missing data and environmental variation. *Sr57* is thought to be present in PI 362698–1 according to the diagnostic markers csLv34 and wMAS000003, however no significant QTLs (α = 0.05) were detected at the *Sr57* locus.

**Table 3:** Significant QTLs (α = 0.05) detected for seedling (HHBJ, HPLB, MCCF, QFCQC, and TRTTF) and adult-plant (Kenya 2014 and Kenya 2015) trials. Positions described are for 95% confidence intervals as estimated via the 2-LOD drop method [53]. LOD scores for each QTL are listed with the generalized R^2^ values in parenthesis

QTL	Position	HHBJ	HPLB	MCCF	QFCQC	TRTTF	Kenya 2014	Kenya 2015
*QSr.ace-2B*	IWB2335 (181.4) - IWB23589 (182.1)				12.4 (0.34)		–	–
*QSr.ace-3B*	IWB68368 (79.7) - IWA4847 (91.3)			15.4 (0.45)				
	IWA4847 (91.3) - IWB25408 (98.1)						17.1 (0.47)	
	IWB25408 (98.1) - IWA5323 (106.6)							15.3 (0.50)
*QSr.ace-6A*	IWB49090 (6.5) - IWB2598 (7.5)					54.1 (0.86)	–	–
	IWB2598 (7.5) - IWB67416 (8.2)			14.6 (0.43)				
*Qsr.ace-6D1*	IWB262 (17.4) - IWB6902 (17.7)	44.4 (0.80)	18.7 (0.50)				–	–
*Qsr.ace-7A2*	IWA1517 (53.3) - IWB9275 (55.3)			15.4 (0.45)			–	–

**Fig. 3:**
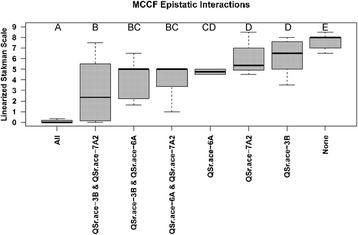
Epistatic interactions of *QSr.ace-3B*, *QSr.ace-6A*, and *QSr.ace-7A2* detected during the MCCF seedling trial. Boxplots represent groups of individuals with different combinations of the three detected QTLs. Groups with the same letters (A-E) are not statistically different (α = 0.05)

The MCCF seedling trial was the only trial where multiple QTLs were detected (Table 3). The individuals within the population were divided into groups based on the presence or absence of the PI 362698–1 allele at markers in the middle of the QTL region defined by the 2-LOD drop method [53]. The Kruskal-Wallis test was performed on these groups and found differences between different QTL combinations. Post-hoc analysis with Student’s T-test identified that the PI 362698–1 allele was required for all three QTLs; *QSr.ace-3B*, *QSr.ace-6A*, and *QSr.ace-7A2*; for the highest level of resistance (Fig. 3). The PI 362698–1 allele is required for at least two QTLs to provide resistance (Fig. 3). If the PI 362698–1 allele is present for only one QTL, the individual is less susceptible than individuals without a PI 362698–1 allele for any of the QTLs (Fig. 3).

## Discussion

Composite interval mapping (CIM) is one of the most popular algorithms used in plant QTL mapping experiments, however its robustness and power is less than that of multiple interval mapping (MIM) [51, 54]. The increased power of MIM stems from the algorithm considering multiple chromosomal regions simultaneously allowing for the detection of multiple QTLs and improved detection of epistatic interactions [54]. Both of these algorithms, as well as simple interval mapping and Bayesian interval mapping, assume that trait data are continuous and normally distributed [51, 53–57]. As such, these algorithms experience reduced power when applied to categorical or non-normally distributed data, which is commonly found in agricultural studies [51, 58, 59]. Data normality is an important factor which is often overlooked for many QTL studies and it can influence the ability to detect minor effect QTLs and partial disease resistance, especially in the presence of large effect QTLs [51, 59]. Transformation of data is an option to meet normality assumptions, but transformation often introduces unwanted biases that skew QTL effect estimates [51]. Algorithms based on a general linearized framework, such as the MIM-GLZ algorithm, are an excellent option to overcome model assumption violations in trait data and provide more power than traditional algorithms used on transformed data [51, 59]. In the present study, the MIM-GLZ algorithm was used to dissect the complex stem rust resistance found in the highly resistant Montenegrin spring wheat landrace PI 362698. Significant stem rust resistance QTLs (α = 0.05) were identified on chromosomes 2B, 3B, 6A, 6D, and 7A. The QTLs identified on chromosomes 3B (*QSr.ace-3B*), 6A (*QSr.ace-6A*), and 6D (*QSr.ace-6D1*) were identified in multiple trials.

*QSr.ace-3B* was identified using MCCF seedling data and the 2014 and 2015 Kenyan field data and explained 45%, 47%, and 50% of the phenotypic variation, respectively. *QSr.ace-3B* was mapped near the marker NB-LRR3, which has been shown to co-segregate with the recessively inherited *Sr12* [32]. *Sr12* has been shown to be ineffective to many races of the Ug99-lineage races at seedling stages [32, 34, 60]. Rouse et al. [34] observed that the appearance of *Sr12* mesothetic phenotype was inconsistent, possibly due to environmental influences. A large degree of variation was observed when mapping *QSr.ace-3B*, which is likely due to environmental variation and the effects of other QTLs in the genome. Hiebert et al. [32] suggested two resistance genes may be present near the *Sr12* locus and the variation observed may support the hypothesis of two resistance genes. At adult-plant stage, *Sr12* provides some resistance to Ug99-lineage races in ‘Thatcher’, especially in the presence of *Sr57* [32, 34, 61]. ‘Thatcher’ and the line RL6058, a ‘Thatcher’ background near isogenic line containing *Sr57*, were included for evaluation during the 2015 Kenyan adult-plant trial. Both ‘Thatcher’ and RL6058 were more resistant than the susceptible line LMPG-6 with median scores of 35MSS and 5MS, respectively. RL6058 has a similar level of severity to PI 362698–1, which is thought to have *Sr57* based on diagnostic markers csLv34 and wMAS000003. However, PI 362698–1 consistently had a lower disease response than RL6058, ranging from R to MR. As such, *QSr.ace-3B* is likely *Sr12* or an allele of *Sr12.*

Marker density near the centromere of 7D is low resulting in two linkage groups. The pleiotropic disease resistance gene *Sr57* mapped to the centromeric end of the 7D2 linkage group. However, no significant QTLs (α = 0.05) near the *Sr57* locus were observed during any of the trials. *Sr57* has been shown to be greatly influenced by environmental variation, which can explain why no effects were detected [32, 34, 47, 62]. *Sr57* is often considered as an adult-plant resistance gene, however it has been shown to enhance the effects of other stem rust resistance genes at the seedling stage [32, 61, 63]. To determine if *Sr57* was confounding the detection of QTLs for mesothetic resistance, a QTL analysis was performed with the TTKSF+ data using a subset of 68 individuals lacking *Sr57. QSr.ace-3B* was detected as significant (α = 0.05) and explained 60.8% of the phenotypic variation*.* The combination of environmental influences on *QSr.ace-3B* and *Sr57*, recessive inheritance, and the segregation distortion observed on the long arm of chromosome 3B may explain the abnormal segregation ratios observed at the F_2_ generation [30]. PI 362698–1 was not an aneuploid based on a chromosome count (data not shown). PI 362698–1 and the RIL population produced some tillers with sterile heads, indicating a degree of infertility. Additionally, RILs often displayed reduced vigor. Therefore, a gene or set of epistatic genes affecting fertility and linked to the areas of segregation distortion may be present in PI 362698–1.

*QSr.ace-6A* was detected with MCCF and TRTTF seedling data and explained 43% and 86% of the phenotypic variation, respectively. The genes *Sr8a*, *Sr22*, *Sr24*, *Sr26*, *Sr27*, *Sr31*, *Sr33*, *Sr35*, *Sr39*, *Sr40*, *Sr46*, *Sr47*, *Sr50*, and *SrSatu* are all effective against TRTTF [8]. Of the effective genes, only *Sr8a* originates in *T. aestivum*, maps to the short arm of chromosome 6A, and provides resistance to both MCCF and TRTTF. Moreover, comparative mapping suggests *QSr.ace-6A* may be *Sr8a* based on the regions identified in ‘Harvest’, SD4279, W7984, and Cltr 15,026, where resistance to TRTTF is thought to be mediated by *Sr8a* or a *Sr8* allele [25, 64–66].

*QSr.ace-6D1* was detected with HHBJ and HPLB seedling data and explained 80% and 50% of the phenotypic variation, respectively. *Sr5*, *Sr42*, *SrCad,* and *SrTmp* map near *QSr.ace-6D1* [67–71]. *Sr42*, *SrCad,* and *SrTmp* all provide resistance to some Ug99-lineage races, however *Sr5* does not [60, 67, 68, 71]. As *QSr.ace-6D1* was not identified from 2014 and 2015 field data, *QSr.ace-6D1* is likely *Sr5*. Moreover, *Sr5* is known to have a characteristically low infection type when challenged with avirulent races, often ranging from ‘0’ to ‘0;’ [72]. This low infection type was observed in PI 362698 when inoculated with races HHBJ, HKHJ, HKQJ, HPCJ, and HPLB, which are known to be avirulent to *Sr5* (Table 1).

*QSr.ace-2B* was detected with QFCQC seedling data and explained 34% of the phenotypic variation. The *T. aestivum*-derived stem rust resistance genes *Sr9, Sr16, Sr28,* and *SrWLR* are present on the long arm of chromosome 2B [14, 28, 40]. Comparative mapping suggests *QSr.ace-2B* is likely *Sr16*, as the QTL maps approximately 30 cM distal of the postulated location of *Sr28* on the LMPG-6/PI 362698–1 map [27, 28, 40]*.* Both *Sr9* and *SrWLR* map approximately 12 cM proximal to *Sr28*, suggesting they cannot be *QSr.ace-2B* [14, 28, 40]. Moreover, *Sr28* provides resistance to the Ug99 lineage races TTKST [27]. As *QSr.ace-2B* was not detected during either of the adult plant trials in Kenya it is unlikely to be *Sr28* and is more likely to be *Sr16*. *Sr16* is thought to be in the background of many wheat accessions and virulence to *Sr16* is relatively common [33].

*QSr.ace-7A2* was detected with MCCF seedling data and explained 45% of the phenotypic variation. Linkage group 7A2 represents the long arm of chromosome 7A. A non-significant (α = 0.10) QTL in this location was observed with the TTKSF+ data when using the subset population. Two resistance genes, *Sr15* and *Sr22*, and two resistance QTL have been reported on the long arm of chromosome 7A [26, 73, 74]. *Sr22* was introgressed into *T. aestivum* from *T. monococcum* L. and provides resistance to Ug99 and TRTTF [8, 26, 60, 73, 74]. As *QSr.ace-7A2* was not detected with the Kenyan or TRTTF data, it is unlikely to be *Sr22* (Table 3)*.* Moreover, *QSr.ace-7A2* maps distally to the postulated location of *Sr22* on the LMPG-6/PI 362698–1 map [26, 42, 75]. QSr*.ace-7A2* maps to the same location as *QSr.abr-*7AL [26], the QTL detected by Pujol et al. [74], and *Sr15* (E. Babiker, personal communication)*.* The Pujol et al. QTL [74] provided resistance to TTKSK and is thought to either be an allele of *Sr15* or a homoeologous allele of a stem rust suppressor thought to be present in ‘Thatcher’ derived cultivars. As such, *QSr.ace-7A2* is likely an allele of *Sr15*, however we are unable to conclude if the QTL is different than the *QSr.abr-7AL* [26] or the Pujol et al. QTL [74].

Newcomb et al. [29] assessed PI 362698 with diagnostic markers associated with the stem rust resistance genes *Sr2, Sr24, Sr31, Sr36*, and *Sr57* and modern breeding associated genes *Rht-B1b*, *Rht-D1b,* and *Ppd-D1a*. The genes *Rht-B1b* and *Rht-D1b* are associated with reduced plant height and *Ppd-D1a* is associated with photoperiod insensitivity which has a large effect on flowering time and heading date [29]. PI 362698 was positive for *Sr57* and *Ppd-D1a*, but negative for *Rht-B1b* and *Rht-D1b* [29]. Despite its short stature, PI 362698 was thought to be a landrace and not the product of modern breeding because *Sr57* and *Ppd-D1a* are thought to have originated multiple times [29]. ‘Thatcher’ is postulated to have *Sr5, Sr12,* and *Sr16* [32, 34, 61]. As QTLs thought to be associated with *Sr5, Sr12*, and *Sr16* were detected in PI 362698–1 it is possible that PI 362698–1 may not be a landrace and may be derived from ‘Thatcher’ (Table 3). To determine the similarity of ‘Thatcher’ and PI 362698–1, the alleles for each were compared at the 6866 markers mapped in the LMPG-6/PI 362698–1 population (Additional file 1: Table S1). ‘Thatcher’ and PI 362698–1 were found to be 76.2% similar at the mapped loci. As such, it is highly likely that ‘Thatcher’ was used as a parent multiple times in the pedigree of PI 362698 and PI 362698 is not a landrace as previously reported [29]. Despite the high similarity ‘Thatcher’ and PI 362698–1 appeared morphologically distinct when grown to maturity in the NDSU green houses.

## Conclusions

The identification of stem rust resistance QTLs at genomic locations with known resistance genes makes PI 362698 a useful accession for future breeding efforts. Five significant QTLs (α = 0.05) were detected on chromosomes 2B, 3B, 6A, 6D, and 7A. The QTL on chromosomes 2B, 3B, 6A, and 6D are thought to be *Sr16*, *Sr12*, *Sr8a*, and *Sr5*, respectively, with Ug99-lineage race resistance being inherited from *Sr12.* Interestingly, *Sr5*, *Sr12*, and *Sr16* are present in the cultivar Thatcher which displays broad resistance to stem rust [32, 34, 61]. In ‘Thatcher’, *Sr12* was inherited from the durum line Iumillo [32]. The stem rust resistance present in PI 362698–1 and ‘Thatcher’ is similar and the two were 76.2% similar genetically suggesting PI 362698 is not a landrace and may be a product of modern breeding efforts with ‘Thatcher’ as an ancestor. The QTL detected on chromosome 7A, *QSr.ace-7A2*, is possibly *Sr15*, *QSr.abr-7AL*, or the QTL detected by Pujol et al. [74]. If *QSr.ace-7A2* is the QTL detected by Pujol et al. [74], it could support the hypothesis that ‘Thatcher’ is an ancestor of PI 362698. Additional work is needed to determine the range of resistance *QSr.ace-7A2*. Despite *Sr57* being present in PI 362698–1, according to diagnostic markers, no QTLs were detected during any of the trials at the *Sr57* locus. Crosses have been made with PI 362698–1 to begin introgressing its stem rust resistance into adapted North Dakota wheat germplasm.

## Additional file

Additional file 1: Table S1. The LMPG-6/PI 362698–1 linkage map and the positions of significant QTLs detected using seedling and adult data. (XLSX 295 kb)

## Abbreviations

CIM: Composite interval mapping
cM: Centimorgans
CTAB: Cetyltrimethylammonium Bromide
MIM: Multiple Interval Mapping
*Pgt*: *Puccinia graminis* f. sp. *tritici*
QTL: Quantitative Trait Locus
RIL: Recombinant Inbred Line

## References

[1] Leonard KJ, Szabo LJ (2005). Stem rust of small grains and grasses caused by *Puccinia graminis*. Mol Plant Pathol.

[2] Roelfs AP, Roelfs AP, Bushnell WR (1985). Wheat and rye stem rust. The cereal rusts vol II: diseases, distribution, epidemiology and control.

[3] Singh RP, Hodson DP, Huerta-Espino J, Jin Y, Bhavani S, Njau P, Herrera-Foessel S, Singh PK, Singh S, Govindan V (2011). The emergence of Ug99 races of the stem rust fungus is a threat to world wheat production. Annu Rev Phytopathol.

[4] Singh RP, Hodson D, Huerta-Espino J, Jin Y, Njau P, Wanyera R, Herrera-Foessel S, Ward RW (2008). Will stem rust destroy the world's wheat crop. Adv Agron.

[5] Singh RP, Hodson DP, Jin Y, Lagudah ES, Ayliffe MA, Bhavani S, Rouse MN, Pretorius ZA, Szabo LJ, Huerta-Espino J, Basnet BR, Lan C, Hovmøller MS (2015). Emergence and spread of new races of wheat stem rust fungus: continued threat to food security and prospects of genetic control. Phytopathology.

[6] Kolmer JA, Jin Y, Long DL (2007). Wheat leaf and stem rust in the United States. Aust J Agric Res.

[7] Visser B, Herselman L, Pretorius ZA (2009). Genetic comparison of Ug99 with selected south African races of *Puccinia graminis* f. sp. *tritici*. Mol Plant Pathol.

[8] Olivera PD, Jin Y, Rouse M, Badebo A, Fetch T, Singh RP, Yahyaoui A (2012). Races of *Puccinia graminis* f. sp. *tritici* with combined virulence to *Sr13* and *Sr9e* in a field stem rust screening nursery in Ethiopia. Plant Dis.

[9] Olivera P, Newcomb M, Szabo LJ, Rouse M, Johnson J, Gale S, Luster DG, Hodson D, Cox JA, Burgin L, Hort M, Gilligan CA, Patpour M, Justesen AF, Hovmøller MS, Woldeab G, Hailu E, Hundie B, Tadesse K, Pumphrey M, Singh RP, Jin Y (2015). Phenotypic and genotypic characterization of race TKTTF of *Puccinia graminis* f. sp. *tritici* that caused a wheat stem rust epidemic in southern Ethiopia in 2013/14. Phytopathology.

[10] Pretorius ZA, Singh RP, Wagoire WW, Payne TS (2000). Detection of virulence to wheat stem rust resistance gene *Sr31* in *Puccinia graminis* f. sp. *tritici* in Uganda. Plant Dis.

[11] Jin Y, Szabo LJ, Pretorius ZA, Singh RP, Ward R, Fetch T (2008). Detection of virulence to resistance gene *Sr24* within race TTKS of *Puccinia graminis* f. sp. *tritici*. Plant Dis.

[12] Patpour M, Hovmøller MS, Justesen AF, Newcomb M, Olivera P, Jin Y, Szabo LJ, Hodson D, Shahin AA, Wanyera R, Habarurema I, Wobibi S (2015). Emergence of virulence to *SrTmp* in the Ug99 race group of wheat stem rust, *Puccinia graminis* f. sp. *tritici*, in Africa. Plant Dis.

[13] Pretorius ZA, Szabo LJ, Boshoff WHP, Herselman L, Visser B (2012). First report of a new TTKSF race of wheat stem rust (*Puccina graminis* f. sp. *tritici*) in South Africa and Zimbabwe. Plant Dis.

[14] Rouse MN, Nirmala J, Jin Y, Chao S, Fetch TG, Pretorius ZA, Hiebert CW (2014). Characterization of *Sr9h*, a wheat stem rust resistance allele effective to Ug99. Theor Appl Genet.

[15] Rouse MN, Wanyera R, Njau P, Jin Y (2011). Sources of resistance to stem rust race Ug99 in spring wheat germplasm. Plant Dis.

[16] Tanksley SD, McCouch SR (1997). Seed banks and molecular maps: unlocking genetic potential from the wild. Science.

[17] Dreisigacker S, Zhang P, Warburton ML, Skovmand B, Hoisington D, Melchinger AE (2005). Genetic diversity among and within CIMMYT wheat landrace accessions investigated with SSRs and implications for plant genetic resources management. Crop Sci.

[18] Reif JC, Zhang P, Dreisigacker S, Warburton ML, van Ginkel M, Hoisington D, Bohn M, Melchinger AE (2005). Wheat genetic diversity trends during domestication and breeding. Theor Appl Genet.

[19] Villa TCC, Maxted N, Scholten M, Ford-Lloyd B (2005). Defining and identifying crop landraces. Plant Genet Resour.

[20] Warburton ML, Crossa J, Franco J, Kazi M, Trethowan R, Rajaram S, Pfeiffer W, Zhang P, Dreisigacker S, van Ginkel M (2006). Bringing wild relatives back into the family: recovering genetic diversity in CIMMYT improved wheat germplasm. Euphytica.

[21] Fu B, Chen Y, Li N, Ma H, Kong Z, Zhang L, Jia H, Ma Z (2013). *pmX*: a recessive powdery mildew resistance gene at the *Pm4* locus identified in wheat landrace Xiaohongpi. Theor Appl Genet.

[22] Gurung S, Mamidi S, Bonman JM, Jackson EW, Del Rio LE, Acevedo M, Mergoum M, Adhikari TB (2011). Identification of novel genomic regions associated with resistance to *Pyrenophora tritici-repentis* races 1 and 5 in spring wheat landraces using association analysis. Theor Appl Genet.

[23] Liu Z, Zurn JD, Kariyawasam G, Faris JD, Shi G, Hansen J, Rasmussen JB, Acevedo M (2017). Inverse gene-for-gene interactions contribute additively to tan spot susceptibility in wheat. Theor Appl Genet.

[24] Xiao M, Song F, Jiao J, Wang X, Xu H, Li H (2013). Identification of the gene *Pm47* on chromosome 7BS conferring resistance to powdery mildew in the Chinese wheat landrace Hongyanglazi. Theor Appl Genet.

[25] Babiker EM, Gordon TC, Bonman JM, Chao S, Rouse MN, Jin Y, Newcomb M, Wanyera R, Bhavani S (2017). Genetic loci conditioning adult plant resistance to the Ug99 race group and seedling resistance to races TRTTF and TTTTF of the stem rust pathogen in wheat landrace Cltr 15026. Plant Dis.

[26] Babiker EM, Gordon TC, Chao S, Newcomb M, Rouse MN, Jin Y, Wanyera R, Acevedo M, Brown-Guedira G, Williamson S, Bonman JM (2015). Mapping resistance to the Ug99 race group of the stem rust pathogen in a spring wheat landrace. Theor Appl Genet.

[27] Babiker EM, Gordon TC, Chao S, Rouse MN, Wanyera R, Acevedo M, Brown-Guedira G, Bonman JM (2017). Molecular mapping of stem rust resistance loci effective against the Ug99 race group of the stem rust pathogen and validation of a single nucleotide polymorphism marker linked to stem rust resistance gene *Sr28*. Plant Dis.

[28] Zurn JD, Newcomb M, Rouse MN, Jin Y, Chao S, Sthapit J, See DR, Wanyera R, Njau P, Bonman JM, Brueggeman R, Acevedo M (2014). High-density mapping of a resistance gene to Ug99 from the Iranian landrace PI 626573. Mol Breeding.

[29] Newcomb M, Acevedo M, Bockelman HE, Brown-Guedira G, Goates B, Jackson EW, Jin Y, Njau PN, Rouse MN, Singh D, Wanyera R, Bonman JM (2013). Field resistance to the Ug99 race group of the stem rust pathogen in spring wheat landraces. Plant Dis.

[30] Acevedo M, Newcomb M, Rouse M, Bockelman HE, Goates BJ, Jackson EW, Jin Y, Brown-Guedira G, Kilian A, Njau P, Singh D, Wanyera R, Bonman JM. Looking for a needle in a haystack: screening of the international stem rust nursery in Kenya for new sources of resistance in spring wheat expo landraces. Minnesota: Borlaug global rust initiative 2011 technical workshop June 13-16 Saint Paul; 2011. p. 140–3.

[31] Stakman EC, Stewart DM, Loegering WQ. Identification of physiologic races of *Puccinia graminis* Var. *tritici*. US Dept Agric, Agric Res Serv. 1962:E-617.

[32] Hiebert CW, Kolmer JA, McCartney CA, Briggs J, Fetch T, Bariana H, Choulet F, Rouse MN, Spielmeyer W (2016). Major gene for field stem rust resistance co-locates with resistance gene *Sr12* in ‘Thatcher’ wheat. PLoS One.

[33] Kolmer JA, Garvin DF, Jin Y (2012). Expression of a Thatcher wheat adult plant stem rust resistance QTL on chromosome arm 2BL is enhanced by *Lr34*. Crop Sci.

[34] Rouse MN, Talbert LE, Singh D, Sherman JD (2014). Complementary epistasis involving *Sr12* explains adult plant resistance to stem rust in Thatcher wheat (*Triticum aestivum* L). Theor Appl Genet.

[35] Knott DR (1990). Near-isogenic lines of wheat carrying genes for stem rust resistance. Crop Sci.

[36] Rowell JB, Bushnell WR, Roelfs AP (1984). Controlled infection by *Puccinia graminis* f. sp. *tritici* under artificial conditions. The cereal rusts, origins, specificity, structure, and physiology, vol 1.

[37] Pretorius ZA, Jin Y, Bender CM, Herselman L, Prins R (2012). Seedling resistance to stem rust race Ug99 and marker analysis for *Sr2*, *Sr24* and *Sr31* in south African wheat cultivars and lines. Euphytica.

[38] Peterson RF, Campbell AB, Hannah AE (1948). A diagrammatic scale for estimating rust intensity of leaves and stem of cereals. Can J Res Sect.

[39] Roelfs AP, Singh RP, Saari EE (1992). Diseases of wheat: concepts and methods of disease management.

[40] Rouse MN, Nava IC, Chao S, Anderson JA, Jin Y (2012). Identification of markers linked to the race Ug99 effective stem rust resistance gene *Sr28* in wheat (*Triticum aestivum* L). Theor Appl Genet.

[41] Stewart N, Via LA (1993). Rapid CTAB DNA isolation technique useful for RAPD fingerprinting and other PCR applications. BioTechniques.

[42] Wang S, Wong D, Forrest K, Allen A, Chao S, Huang BE, Maccaferri M, Salvi S, Milner SG, Catticelli L, Mastrangelo AM, Whan A, Stephen S, Barker G, Wieseke R, Plieske J, Lillemo M, Mather D, Appels R, Dolferus R, Brown-Guedira G, Korol A, Akhunova AR, Feuillet C, Salse J, Morgante M, Pozniak C, Luo M, Dvorak J, Morell M, Dubcovsky J, Ganal M, Tuberosa R, Lawley C, Mikoulitch I, Cavanagh C, Edwards KJ, Hayden M, Akhunov E, International Wheat Genome Sequencing Consortium (2014). Characterization of polyploid wheat genomic diversity using a high density 90 000 single nucleotide polymorphism array. Plant Biotechnol J.

[43] Lagudah ES, McFadden H, Singh RP, Huerta-Espino J, Bariana HS, Spielmeyer W (2006). Molecular genetic characterization of the *Lr34/Yr18* slow rusting resistance gene region in wheat. Theor Appl Genet.

[44] Brown-Guedira G, Dreisigacker S (2013). MAS data.

[45] Lorieux M (2012). MapDisto: fast and efficient computation of genetic linkage maps. Mol Breeding.

[46] Kosambi DD (1944). The estimation of map distances from recombination values. Ann Eugenics.

[47] Yu L, Lorenz A, Rutkoski J, Singh RP, Bhavani S, Huerta-Espino J, Sorrells M (2011). Association mapping and gene-gene interaction for stem rust resistance in CIMMYT spring wheat germplasm. Theor Appl Genet.

[48] Zhang D, Bowden RL, Yu J, Carver BF, Bai G. Association analysis of stem rust resistance in U.S. winter wheat. PLoS One. 2014; 10.1371/journal.pone.0103747. 10.1371/journal.pone.0103747 PMC4114971 25072699

[49] R Core Team (2016). R: a language and environment for statistical computing.

[50] Joehanes R, Nelson JC (2008). QGene 4.0, an extensible java QTL-analysis platform. Bioinformatics.

[51] Joehanes R (2009). QTL mapping methods based on the generalized linear model. Generalized and multiple-trait extensions to quantitative-trait locus mapping. Kansas State University, Manhattan, KS, U.S.a.

[52] Churchill GA, Doerge RW (1994). Empirical threshold values for quantitative trait mapping. Genetics.

[53] Lander ES, Botstein D (1989). Mapping mendelian factors underlying quantitative traits using RFLP linkage maps. Genetics.

[54] Kao C, Zeng Z, Teasdale D (1999). Multiple interval mapping for quantitative trait loci. Genetics.

[55] Haley CS, Knott SAA (1992). Simple regression method for mapping quantitative trait loci in line crosses using flanking markers. Heredity.

[56] Stephens DA, Fisch RD (1998). Bayesian analysis of quantitative trait locus data using reversible jump Markov chain Monte Carlo. Biometrics.

[57] Zeng ZB (1994). Precision mapping of quantitative trait loci. Genetics.

[58] Box GEP (1953). Norn-normality and tests on variances. Biometrika.

[59] Dahleen LS, Morgan W, Mittal S, Bregitzer P, Brown RH, Hill N (2012). Quantitative trait loci (QTL) for *Fusarium* ELISA compared to QTL for Fusarium head blight resistance and deoxynivalenol content in barley. Plant Breed.

[60] Jin Y, Singh RP, Ward RW, Wanyera R, Kinyua M, Njau P, Fetch T, Pretorius ZA, Yahyaoui A (2007). Characterization of seedling infection types and adult plant infection responses of monogenic *Sr* gene lines to race TTKS of *Puccinia graminis* f. sp. *tritici*. Plant Dis.

[61] Gavin Vanegas CD, Garvin DF, Kolmer JA (2008). Genetics of stem rust resistance in the spring wheat cultivar Thatcher and the enhancement of stem rust resistance by *Lr34*. Euphytica.

[62] Rutkoski JE, Poland JA, Singh RP, Huerta-Spino J, Bhavani S, Barbier H, Rouse MN, Jannink J, Sorrells ME (2014). Genomic selection for quantitative adult plant stem rust resistance in wheat. Plant Genome.

[63] Kerber ER, Aung T (1999). Leaf rust resistance gene *Lr34* associated with nonsuppression of stem rust resistance in the wheat cultivar Canthatch. Phytopathology.

[64] Dunckel SM, Olson EL, Rouse MN, Bowden RL, Poland JA (2015). Genetic mapping of race-specific stem rust resistance in the synthetic hexaploid W7984 × Opata M85 mapping population. Crop Sci.

[65] Guerrero-Chavez R, Glover KD, Rouse MN, Gonzalez-Hernandez JL (2015). Mapping of two loci conferring resistance to wheat stem rust pathogen races TTKSK (Ug99) and TRTTF in the elite hard red spring wheat line SD4279. Mol. Breeding..

[66] Hiebert CW, Rouse MN, Nirmala J, Fetch T (2017). Genetic mapping of stem rust resistance to *Puccinia graminis* f. sp. *tritici* race TRTTF in the Canadian wheat cultivar harvest. Plant Dis.

[67] Hiebert CW, Fetch TG, Zegeye T, Thomas JB, Somers DJ, Humphreys DG, McCallum BD, Cloutier S, Singh D, Knott DR (2011). Genetics and mapping of seedling resistance to Ug99 stem rust in Canadian wheat cultivars ‘peace’ and ‘AC Cadillac. Theor Appl Genet.

[68] Hiebert CW, Kassa MT, McCartney CA, You FM, Rouse MN, Fobert P, Fetch TG (2016). Genetics and mapping of seedling resistance to Ug99 stem rust in winter wheat cultivar triumph 64 and differentiation of *SrTmp, SrCad,* and *Sr42*. Theor Appl Genet.

[69] Ghazvini H, Hiebert CW, Zegeye T, Liu S, Dilawari M, Tsilo T, Anderson JA, Rouse MN, Jin Y, Fetch T (2012). Inheritance of resistance to Ug99 stem rust in wheat cultivar Norin 40 and genetic mapping of *Sr42*. Theor Appl Genet.

[70] Gao L, Kielsmeier-Cook J, Bajgain P, Zhang X, Chao S, Rouse MN, Anderson JA (2015). Development of genotyping by sequencing (GBS)- and array-derived SNP markers for stem rust resistance gene *Sr42*. Mol Breeding.

[71] Kassa MT, You FM, Fetch TG, Fobert P, Sharpe A, Pozniak CJ, Menzies JG, Jordan MC, Humphreys G, Zhu T, Luo M, McCartney CA, Hiebert CW (2016). Genetic mapping of *SrCad* and SNP marker development for marker-assisted selection of Ug99 stem rust resistance in wheat. Theor Appl Genet.

[72] Luig NH, Rajaram S (1972). The effect of temperature and genetic background on host gene expression and interaction to *Puccinia graminis tritici*. Phytopathology.

[73] Olson E, Brown-Guedira G, Marshall D, Stack E, Bowden RL, Jin Y, Rouse M, Pumphrey MO (2010). Development of wheat lines having a small introgressed segment carrying stem rust resistance gene *Sr22*. Crop Sci.

[74] Pujol V, Forrest KL, Zhang P, Rouse MN, Hayden MJ, Huang L, Tabe L, Lagudah E (2015). Identification of a stem rust resistance locus effective against Ug99 on wheat chromosome 7AL using a RAD-Seq approach. Theor Appl Genet.

[75] Yu L, Barbier H, Rouse MN, Singh S, Singh RP, Bhavani S, Huerta-Espino J, Sorrells MEA (2014). Consensus map for Ug99 stem rust resistance loci in wheat. Theor Appl Genet.

